# Society Meetings

**Published:** 1913-03

**Authors:** 


### SOCIETY MEETINGS

---

THE BUFFALO ACADEMY OF MEDICINE gave a dinner to Dr. Ramon Guiteras of New York, February 5, at the Statler. At the meeting which followed, Dr. Guiteras gave a lantern demonstration of prostatic work, of which he has promised a full abstract for publication in the BUFFALO MEDICAL JOURNAL. Dr. Nelson W. Wilson gave a paper on "Conservatism in Prostatic Operations."

---

A SOCIETY FOR THE ADVANCEMENT OF CLINICAL STUDY has been organized in N. Y. City, to furnish information to resident and visiting physicians regarding clinics, etc. A bureau has been established at the Academy of Medicine, 19 W. 43d St., with a clerk on duty from 9 to 6 to answer telephone calls (Bryant 974.) A bulletin board for announcement of standing clinics and special operations scheduled for each day has also been provided. The BUFFALO MEDICAL JOURNAL will publish announcements from time to time and, if such announcements are not regularly printed, will keep them on file for reference. Inquiry should be made of the editor before 1 P. M.

---

THE INTERNATIONAL CONGRESS OF OPHTHALMOLOGY will meet in St. Petersburg August 10-15 (July 28- August 2, O. S. still in use in Russia) 1914 (not 1913). Inscription fee 25 francs (about $5.00) for members and 10 francs for members of their families. Cards will be issued which will secure admission to museums, reduced railroad fares and other privileges. Prof. L. G. Bellarminoff is President of the Central Bureau and of the International Organization Committee. For further information, registration, etc., address Dr. Th. Germann, Secretary, Ophthalmic Hospital, St. Petersburg, Mochowaja 38.

---

THE SECOND INTERNATIONAL CONGRESS OF LIFE SAVING AND PREVENTION OF ACCIDENTS will meet in Vienna, September 9-13, 1913. Membership fee 25 Kronen ($5.00) ; fee for members of family 15 Kronen ($3.00). Money orders should be sent to the Treasurer, Herr kaiserlicher Rat, Johann Thomas Wancura, Chef des Bankhauses Schelhammer & Schattera Stefansplatz 11, Vienna.

---

THE CHICAGO MEDICAL SOCIETY gave a banquet in honor of its ex-Presidents, at the Hotel Sherman, February 12. All twenty of the living ex-presidents were scheduled for speeches.

---

THE HOMOEOPATHIC MEDICAL SOCIETY OF THE STATE OF NEW YORK held its annual meeting in Albany early in February. Officers were elected as follows: President, B. W. Sherwood of Syracuse; Secretary, Bert B. Clark of N. Y.; Treasurer, Reeve B. Howland of Elmira; Necrologist, John L. Moffatt of Ithaca. The society endorsed Dr. Eugene H. Porter for reappointment as State Commissioner of Health.

THE BUFFALO ACADEMY OF MEDICINE, Section of Pathology, met February 25, after a subscription dinner to Dr. Frederic L. Sondern of N. Y., who presented "The Value of Blood Examinations in Acute Inflammatory Lesions. Dr. John Eckel gave a paper on "Serum Reactions in Diseases of the Nervous System."

The regular meeting of the MEDICAL SOCIETY OF THE COUNTY OF ERIE was held on Monday, February 17th, 1913, and was called to order by President Whitwell at 9 P. M.

Minutes of the annual meeting and those of the Council meetings were read and approved.

Dr. F. Park Lewis, Chairman Committee on Legislation, outlined a model bill drawn by the Legislative Committee of the American Medical Association, which will be presented to the Association at its next meeting, and, if adopted, will be submitted to the various State legislatures. This bill has for its object the prevention of blindness by dealing with ophthalmia neonatorum. He also submitted the bill now before the New York State Legislature, requiring medical certificates before marriage licenses are issued. This bill was approved by the Council and confirmed by the Society by adopting the Council's minutes.

Dr. Lewis gave the history of a child, born with perfect eyes, but which became totally blind from ophthalmia neonatorum.

The question was discussed by Dr. Hopkins, who thought the committee did not go far enough. Dr. Woodruff thought education was preferable to legislation. Dr. VanPeyma said that other germs than the gonococcus caused ophthalmia. Dr. Lewis explained that from thirty to forty per cent. of cases of ophthalmia neonatorum are not due to gonococcus and considered it wise not to go into that phase by legislation, as it would cast a stigma on the child's parentage and make reporting of ophthalmia difficult.

The following were elected to membership:

Dr. Paul H. Sandresky, Dr. Herman F. May, Dr. Elmer A. D. Clarke, Dr. E. A. Southall and Dr. A. C. Callahan, the two latter having overlooked the payment of their annual dues, made good and were reinstated.

Dr. Clayton Greene, Secretary of the County Milk Commission, made a verbal report on the work accomplished.

The scientific program consisted of a paper on "A Case of Spastic Paraplegia; results of section of the posterior roots of the spinal cord for the pain and spasticity," by Dr. Prescott Le Breton; "Differential Diagnosis of Dysuria," by Dr. Charles W. Bethune.

FRANKLIN C. GRAM, Secretary.

---

The regular meeting of the ROCHESTER PATHOLOGICAL SOCIETY was held February 20. The program included a demonstration of the Pulmotor by Dr. Walter A. Callahan and a paper on "Multiple Neuritis" by Dr. Edward B. Angell.

The Tompkins County Medical Society held its eighth annual banquet February 25. Dr. Robert T. Morris of New York was the principal guest of honor and gave a paper on "Instances of Colon Bacillus Infection." Rev. Nathaniel Schmidt of Ithaca spoke on "Old Babylonian and Egyptian Medicine."

This County Society was founded in 1818 and continued until 1844, when it disbanded. It was reorganized in 1862 and continued to 1900. A County Association was formed in 1903 and continued to 1906, when its members and the members of the defunct County Society joined forces in a reunited society.

The annual banquet has become a permanent feature for February, as has the joint outing with our adjoining Cortland County Medical Society, for May.

---

The Hospital Medical Society of Rochester met February 13. Dr. H. L. Prince read a paper, "Congenital Dislocation of the Hip."

---

Section II.—(Surgery, including Anatomy, Orthopedic Surgery, Ophthalmology, Otology, Larynology, Dermatology and Genito-Urinary Surgery) of the Rochester Academy of Medicine met at the Academy Rooms, February 12. A paper, "An Adventure in Practice," was given by Dr. J. G. Mumford, Clifton Springs Sanitarium. A subscription dinner was given to Dr. J. G. Mumford at the Rochester Club.

---

Dr. Fred A. Mendlein was the essayist of the February meeting of the Medical and Surgical League of Buffalo. A very excellent paper was read by him entitled, "Some Points in the Etiology and Diagnosis of Dyspepsia."

---

Program for Elmira Academy of Medicine for March 5th: "Benefits to be Derived from Medical Inspection of Schools," Dr. G. M. Case; "Pruritis Ani," Dr. A. H. Baker.

